# Towards safer, better healthcare: harnessing the natural properties of complex sociotechnical systems

**DOI:** 10.1136/qshc.2007.023317

**Published:** 2009-01-26

**Authors:** J Braithwaite, W B Runciman, A F Merry

**Affiliations:** 1 Institute of Health Innovation, University of New South Wales, Sydney, Australia; 2 Centre for Clinical Governance Research in Health, University of New South Wales, Sydney, Australia; 3 School of Public Health and Community Medicine, University of New South Wales, Sydney, Australia; 4 Royal Adelaide Hospital, Joanna Briggs Institute and University of Adelaide, Adelaide, Australia; 5 Australian Patient Safety Foundation, Adelaide, Australia; 6 University of Auckland, Auckland, New Zealand; 7 Quality and Safety Committee of the World Federation of Societies of Anaesthesiologists, London, UK

## Abstract

**Objectives:**

To sustain an argument that harnessing the natural properties of sociotechnical systems is necessary to promote safer, better healthcare.

**Methods:**

Triangulated analyses of discrete literature sources, particularly drawing on those from mathematics, sociology, marketing science and psychology.

**Results:**

Progress involves the use of natural networks and exploiting features such as their scale-free and small world nature, as well as characteristics of group dynamics like natural appeal (stickiness) and propagation (tipping points). The agenda for change should be set by prioritising problems in natural categories, addressed by groups who self select on the basis of their natural interest in the areas in question, and who set clinical standards and develop tools, the use of which should be monitored by peers. This approach will facilitate the evidence-based practice that most agree is now overdue, but which has not yet been realised by the application of conventional methods.

**Conclusion:**

A key to health system transformation may lie under-recognised under our noses, and involves exploiting the naturally-occurring characteristics of complex systems. Current strategies to address healthcare problems are insufficient. Clinicians work best when their expertise is mobilised, and they flourish in groupings of their own interests and preference. Being invited, empowered and nurtured rather than directed, micro-managed and controlled through a hierarchy is preferable.

An important question facing contemporary health systems is how to reduce variability and iatrogenic harm.^1^ Many initiatives have been proposed in the belief that this is primarily a structural issue.^2^ ^3^ They have taken the form of reorganisation^4^ and policy reforms,^5^ credentialling and accreditation,^6^ ^7^ and directives to standardise care processes.^8^ ^9^ However, at the heart of healthcare lie interactions between individual patients and clinicians. Improving communication and relationships, enhancing individual decision-making through evidence-based decision support^10^ and promoting patients’ involvement in and responsibility for their own care are also vital for safer, better care.

We propose that stimulating the fundamental transformation needed to improve the safety and quality of healthcare will require harnessing the natural properties which emerge (often spontaneously) at the interface between the socio (human behavioural) and technical components of complex systems.^11^ A bottom-up strategy led by clinicians is badly needed to balance the predominantly top-down approaches which frequently result in only modest improvements which are difficult to sustain.^12^ Patient safety is what social scientists call a “wicked problem”—one that is messy, persistent and multidimensional.^13^ ^14^ Politicians and bureaucrats seek to shape clinical practice by edict, whereas in reality it is shaped by the behaviours and attitudes of practising clinicians.

## NATURAL PROPERTIES OF COMPLEX SYSTEMS

Many complex systems have similar natural properties and behaviours (table 1); these have been identified in fields as diverse as mathematics,^15^ sociology,^16^ marketing science^17^ and psychology,^18^ and attempts made to apply them to healthcare.^19^ ^20^ Safety is a property which depends on good organisation, tools and infrastructure as well as on the behaviours of individuals (often in teams). The collective values and behaviours of these individuals comprise the culture of the system. Harnessing their undoubted industry, goodwill and energy, and supporting the natural processes by which they interact and cooperate, rather than constantly reorganising them, is the key to changing this culture.^21^ ^22^ The emphasis should be on guiding the natural properties and behaviours of sociotechnical systems (see table 1) rather than imposing hierarchical structures and above-down instructions from people who do not actually work at the “coal-face.”

**Table 1:** Natural properties and features of complex systems

Properties of complex systems	Healthcare manifestations
Natural networks	Groups of clinicians who interact professionally to share information, support, consult, refer and jointly manage patients
Natural hubs and scale-free behaviour	Opinion leaders in networks who disproportionately influence policies, events or practices
Natural pathways, connectivity and small worlds	Communication channels facilitating the rapid dissemination of information via “grapevines” and communities of practice
Natural appeal and stickiness	Messages and communications that are convincing and are absorbed among clinical cohorts
Natural propagation and tipping points	The point at which a message, idea or practice whose time has come is readily adopted by a critical mass of clinicians
Natural categories and natural mapping	The identification of clinically relevant problems grouped as accessible data, to facilitate decision-making and solutions to healthcare problems
Natural interest and self-selection	Clinicians with common concerns and complementary expertise voluntarily grouped together to collectively resolve coal-face clinical problems

## ARTICULATING THESE NATURAL PROPERTIES

### Natural networks

There are two types of network: those that are purpose-designed, funded and imposed by someone in authority (mandated networks), and those that are formed by relationships among clinicians which rest on mutual (often implicit) agreements to participate (natural networks).^23^ The former have assigned functions, and are necessary and appropriate for the “hotel,” logistic and infrastructure requirements of healthcare. The latter underpin the health system’s purpose: to enhance health and deliver care.

Natural networks respond poorly or not at all to conventional management or control measures. They emerge spontaneously and function with little or no externally imposed structure, but can exert powerful and pervasive influences on how systems actually perform and function, at “street level,” behind the formal organisational charts. They are webs of humans connected personally or via technologies, interacting in multiple ways. With the internet, natural networks of patients and their supporters are also increasing.^24^

**Figure 1:**
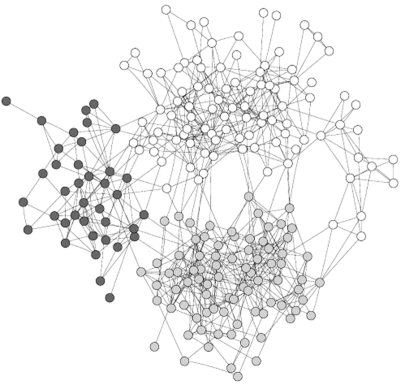
Friendship clusters in a US school. Reprinted with permission from Moody J. Peer influence groups: identifying dense clusters in large networks. *Soc Netw* 2001;**23**:261–83.

Natural networks can be relatively simple or highly complicated,^25^^–^27 and individuals may tap into as many networks as there are problems to be solved or ends to be achieved. They occur wherever humans cluster in the pursuit of a common purpose or activity, such as adolescent friendships in schools (see fig 1),^28^ the sharing of wisdom across boundaries^29^ ^30^ or the improvement of patient care.^31^

Medical examples range from geographically dispersed clinicians weakly related by discipline or common interests (for example, in stroke, trauma or ophthalmology), collaborating to improve care in Australia,^31^ to teams in America reducing catheter-related infections in 103 ICUs,^32^ to action-orientated researchers promoting high levels of cooperation among paediatric clinicians in Finland.^33^

### Natural hubs and scale-free behaviour

**Figure 2:**
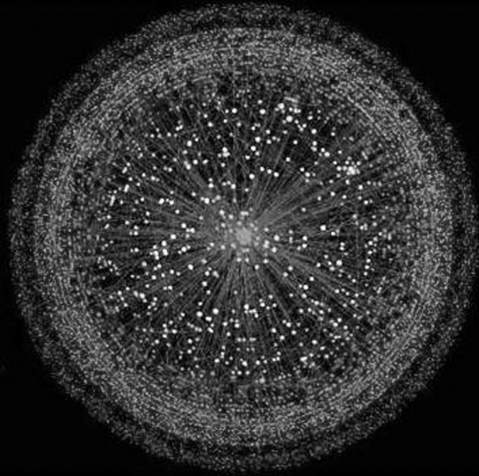
Structural typology of the internet. Reprinted with permission from Carmi S, Havlin S, Kirkpatrick S, *et al*. A model of Internet typology using k-shell decomposition. *Proc Natl Acad Sci U S A* 2007;**104**:11150–4.

Nodes in a network might be thought to be randomly related, each with a similar number of connections. However, most networks are actually “scale-free,” with unequally distributed nodes with varying numbers of connections.^34^^–^36 A few nodes with many connections form “hubs” which emerge naturally and become the distributed force field of the network (for example, the Google search engine, King’s Cross station or a prominent, influential clinician). Scale-free networks are less likely to become congested because they concentrate effort efficiently.^37^ The internet has such a structure, with a limited number of hubs, an inner layer of strongly peer-connected components and an outer layer with many relatively isolated nodes (fig 2).^38^ Analogous patterns emerge in healthcare, where hubs (prestigious services or clinicians) can have pervasive influences on practices and attitudes among their well-linked inner layer colleagues, and progressively less on the outer layer of poorly connected isolates.

### Natural pathways, connectivity and small worlds

Scholars have sought recently to understand the sociology of different types of networks through an analysis of another natural characteristic, that of “small worlds.”^39^ Regardless of the size of any particular network, there are fast routes through them, creating small worlds within large networks.^40^ Even among the six billion people on Earth, for example, we can usually map the ties of any two through substantially less than the famous six degrees of separation.^41^ Making a small world work using natural pathways does not require personal or corporate knowledge of everyone in a chain or explicit representation of connections, pathways or hierarchies.

### Natural appeal and stickiness

Administrators are always trying to spread messages or influence people through directives, emails and meetings. However, most communications are ignored, and meetings avoided or attended only by a few. This brings us to another concept—not a natural feature of networks, but a phenomenon related to group behaviour. It comes from marketing, and is known as stickiness. Any message to be both remembered and acted upon needs to be sticky.^42^ ^43^ Stickiness is a function of the intrinsic nature of a message, how it is presented and the effect it has on the recipient. Sticky messages have natural appeal. Awareness of the importance of stickiness challenges communicators to communicate well. Novel or effective communication, smooth transmission modes, embedded cues in the environment and workplace to remind people of the message, forcing functions to facilitate compliance with the message and a critical mass of champions or opinion leaders can all be important in getting a message to stick.

### Natural propagation and tipping points

The stage at which a critical mass for sustained or escalating momentum in any system is reached is what Gladwell calls a “tipping point,”^18^ an idea used in classic epidemiology. It is the pivotal juncture at which a concept, social movement or epidemic takes hold.^44^ ^45^ One or more triggers may be needed for a state change. Although there are many potential candidates, several stand out: persuasive, catalytic individuals,^46^ memorable, even irresistible messages^47^ and conducive contexts.^45^ A tipping point is not easy to evoke. An example in medicine that has taxed the best minds over the last two decades is “evidence-based medicine,”^48^ an undeniably seductive concept, but one which is yet to demonstrate self-propagation and has proved resistant to conventional measures for inducing change. The missing ingredients for the requisite degree of stickiness may be the redesign of the interface between information technology and clinicians at the point of care, work-flow forcing functions making it easier to do the right thing and harder to do the wrong thing,^49^ and peer-group self-regulation involving meaningful surveillance between colleagues.^50^

**Figure 3:**
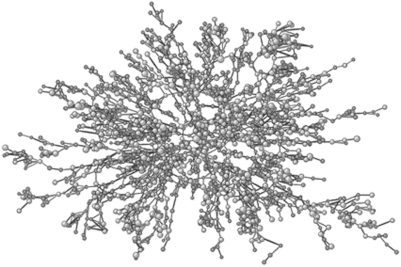
Framingham Heart Study and obesity networks. Reprinted with permission from Christakis N, Fowler J. The spread of obesity in a large social network over 32 years *N Engl J Med* 2007;**357**:370–9.

Examples of the power of networks continue to emerge. In the longstanding Framingham Heart Study, clusters of obese individuals were identified in networks of social ties (fig 3). The risk of obesity for those with obese friends was increased more than 50%. The influential factor was social, not geographic, distance.^51^ The lesson is a profound reminder that desirable or undesirable behaviours can propagate via networks.

### Natural categories and natural mapping

Patients may be placed at avoidable risk by being subjected to a flawed plan, by the flawed execution of a plan or both.^52^ To devise and disseminate effective preventive and corrective strategies, it is necessary to identify what is going wrong and then how and why.^53^ The major studies on appropriateness of care and on adverse events have not adequately provided this information in a manner that is meaningful for clinicians. Qualitative analysis of the problems identified in these studies is needed. They should be placed into clinically meaningful categories (principal natural categories or groups of problems amenable to a common solution), based on their contextual features and contributing factors.^54^ The characteristics of natural categories, with examples, have been described elsewhere.^55^ Natural categories will form the basis of the International Classification for Patient Safety, which is currently being developed.^56^

Natural mapping, a concept described by Norman, allows the components and attributes of an entity, schema or problem to be grouped in a representation reflecting their relationships in the real world.^49^ In healthcare, this facilitates a hierarchical classification of incidents which allows detailed information to be elicited from reports or documents in a comprehensive, structured but intuitive way, and stored in a database from which meaningful information can readily be extracted and analysed for the generation of solutions to problems with safety and patient care.^53^ ^57^ The process includes seeking consensus from groups of clinicians on what the relevant constructs and concepts are, what terms or descriptions should be used and how they should be represented. Testing these constructs and concepts against real-world information (such as incident reports, complaints or coroners’ recommendations) permits iterative improvement, progressively enhancing their validity.^58^ The reliability of these processes is a function of the reproducibility with which incidents can be deconstructed into their components. This in turn depends on how intuitive (or natural) the categories and cascades of questions (maps) are, and how “user-friendly” the overall system is.

## TOWARDS A BOTTOM-UP SOLUTION TO PATIENT SAFETY

### Putting natural interest and self selection to work

For every healthcare problem there are networks, hubs and subclusters made up of clinicians with a special interest and expertise in that area. Such people are typically willing to devote time and effort to solving the problems in which they have a natural interest, as part of their professional lives, whether or not adequate funding is available. “Hub” clinicians should be asked to collaborate with others who have a genuine aptitude and passion for addressing a particular problem, to review that problem, devise and implement corrective measures, and ensure sustained surveillance and frequent updating of the solutions (see box 1).^57^ ^59^ ^60^

**Box 1 A tale of a natural network and propagation of a sticky idea^57^ ^59^**

By the late 1980s, important technological advances provided the potential for safer anaesthesia with devices such as pulse oximeters and capnographs. When anaesthetists requested access to these, they were ignored by administrators. Frustrated, a meeting was called in Australia of influential clinicians. The idea had such natural appeal that 63 of 65 people invited attended the meeting in May 1987, the majority of whom represented hubs of natural networks interested in subjects allied to safety and monitoring. All had to pay their way to the meeting which was called at short notice and not under the auspices of any professional college, society or association. Everyone who was asked to present a paper did so and provided copy for the 36 manuscripts which were published 10 months later.^57^ These recommended minimum standards which were endorsed by the relevant professional bodies at national, and, later, international levels. Despite opposition from administrators, oximeters were introduced for every anaesthetised patient and capnographs for every intubated and/or ventilated patient. The idea “tipped,” and it became unacceptable to conduct anaesthesia in developed countries without the use of these devices.

An analysis of 4000 incidents and 1200 medicolegal reports over the period 2000–2005 revealed not a single case of hypoxic brain damage or death due to inadequate ventilation or undetected oesophageal intubation, problems which had plagued anaesthesia until the late 1980s.^59^ This is an example of quadruple-loop learning (at personal, organisational, national and international levels),^53^ and a bottom-up initiative which gained traction by harnessing some of the natural properties of a sociotechnical system.

### Exploring the proposition

We suggest, in parallel with above-down initiatives to improve the safety and quality of healthcare, that clinical standards be set by expert groups for each of the individual problems which compromise the safety and quality of healthcare by harnessing the natural properties of networks and clinicians’ behaviour. A good start has been made with the development of evidence-based clinical guidelines by organisations such as the National Institute for Health and Clinical Excellence in the UK (NICE)^61^ and the Joanna Briggs Institute (another self-propagating network; see box 2),^62^ but ongoing effort is essential, as new problems will continue to emerge at the leading edge of medical advances. The plethora of new errors associated with the introduction of computer order entry provide an example.^63^ Problems to address initially could be those clearly shown to be widespread, costly and amenable to mitigation by proven interventions of reasonable cost– and risk–benefit.^54^ ^64^ ^65^

**Box 2 Joanna Briggs Institute for Evidence-Based Healthcare—another self-propagating network**

This organisation was formed in 1996 with a single corporate member. It was not under the auspices of any professional body but has alliances with several as well as with hospitals and universities. Within a decade it had 26 centres in 15 countries, with members in 48 countries, and 2000 corporate members. The core business is the generation of annually updated evidence-based guidelines based on systematic literature reviews. There are now 1200 systematic reviews and 200 packages of guidelines which can be compiled, using software available for members, into customised clinical practice manuals. Software is also available for clinical audit, benchmarking and tracking evidence of practice change. There are now plans to harness the concepts and networks to complement international and national collaborations for translating evidence into practice.

Once a problem that needs to be addressed has been identified, a meeting should be arranged to develop a tool to address it. Modest funding should be provided for two or three acknowledged experts to attend and present reviews of the relevant information. A public invitation should be issued to all with an interest in the area. In this way, the natural hubs of existing networks relevant to the problem can be gathered together to strengthen existing links between networks and establish new ones, thereby facilitating the ongoing generation of effective solutions to the particular problem.

Ideal tools should: explain the underlying principles; outline the necessary tasks; record that they have been done; facilitate audit; and promote dissemination, stickiness and use of the principles in question. They should be endorsed by the relevant professional organisations as clinical standards, with a requirement that they should be used whenever appropriate. Their design should make compliance easy, and clinicians should be involved in their design to facilitate this. Documented justification of non-compliance should be required. The underlying rationale for the tool, and the process by which it was developed, should be published (ideally in the peer-review literature) and made available on the internet; a plain language version should be developed for patients and lay-carers. The tool should be refined over time on the basis of feedback from those who use it.

### Natural surveillance and collegial behaviour

A transparent process is needed for identifying persistent non-compliance with these tools, with a view to progressively improving standards. It has been shown that formal surveillance may be acceptable to practising clinicians.^66^ ^67^ We recommend a peer-review audit. This is practical because much routine clinical practice involves the following of pathways and algorithms by individual clinicians through habit, so it is possible to identify the routine practices of most clinicians by reviewing as few as 10 or 20 medical records. We propose that peers review each other’s compliance with agreed tools on a regular basis, as part of normal professional practice, and accreditation agencies can then randomly check for evidence of tool use. This would provide a transparent process for identifying persistent non-compliance with standards and an objective basis for opening discussions to understand and address the issues.

## CONCLUSION

There is now compelling evidence and widespread acceptance that there are problems in healthcare that must be addressed and that current approaches to addressing them are inadequate.^68^ Education, persuasion and attempts to change practice through existing hierarchical structures have largely failed in the face of the entrenched opposing forces of clinical autonomy and the traditional tolerance of individual and regional variations in practice.^11^ Evidence-based practice is overdue. It is time to do something different.

We recommend harnessing the natural properties of the sociotechnical system that comprises healthcare, and guiding the existing practices which come naturally to clinicians, so that these can be developed and used to promote continuous quality improvement with effective self-regulation.^50^ Clinicians, like other professionals, work best when they are allowed to flourish in groupings of their own interests and preference, are empowered rather than directed, and nurtured and influenced by their peers rather than controlled by others. They are likely to become more involved in promoting safer and better care if invited rather than compelled, and should be encouraged to solve naturally occurring problems in voluntary collaborations with their fellow clinicians. To underpin this, we propose facilitating the development and application of clinical standards by means of relevant and effective tools.
